# The Dynamic Changes of AFP From Baseline to Recurrence as an Excellent Prognostic Factor of Hepatocellular Carcinoma After Locoregional Therapy: A 5-Year Prospective Cohort Study

**DOI:** 10.3389/fonc.2021.756363

**Published:** 2021-12-16

**Authors:** Qi Wang, Biyu Liu, Wenying Qiao, Jianjun Li, Chunwang Yuan, Jiang Long, Caixia Hu, Chaoran Zang, Jiasheng Zheng, Yonghong Zhang

**Affiliations:** ^1^ Research Center for Biomedical Resources, Beijing You’an Hospital, Capital Medical University, Beijing, China; ^2^ Interventional Therapy Center for Oncology, Beijing You’an Hospital, Capital Medical University, Beijing, China

**Keywords:** hepatocellular carcinoma (HCC), alpha fetoprotein (AFP), locoregional therapy (LRT), prognosis (carcinoma), prospective

## Abstract

**Background:**

Although many studies have confirmed the prognostic value of preoperative alpha-fetoprotein (AFP) in patients with hepatocellular carcinoma (HCC), the association between AFP at baseline (b-AFP), subsequent AFP at relapse (r-AFP), and AFP alteration and overall survival in HCC patients receiving locoregional therapy has rarely been systematically elucidated.

**Patients and Methods:**

A total of 583 subjects with newly diagnosis of virus-related HCC who were admitted to Beijing You ‘an Hospital, Capital Medical University from January 1, 2012 to December 31, 2016 were prospectively enrolled. The influence of b-AFP, subsequent r-AFP, and AFP alteration on relapse and post-recurrence survival were analyzed.

**Results:**

By the end of follow-up, a total of 431 (73.9%) patients relapsed and 200 (34.3%) died. Patients with positive b-AFP had a 24% increased risk of recurrence compared with those who were negative. Patients with positive r-AFP had a 68% increased risk of death after relapse compared with those who were negative. The cumulative recurrence-death survival (RDS) rates for 1, 3, 5 years in patients with negative r-AFP were 85.6% (184/215), 70.2%(151/215), and 67.4%(145/215), while the corresponding rates were 75.1% (154/205), 51.2% (105/205), and 48.8% (100/205) in those with positive AFP (P<0.001). 35 (21.6%) of the 162 patients with negative b-AFP turned positive at the time of recurrence, and of this subset, only 12 (34.3%) survived. Of the 255 patients with positive b-AFP, 86 (33.7%) turned negative at the time of relapse, and of this subset, only 30 (34.9%) died. The 1-, 3-, and 5-year cumulative RDS rates were also compared among groups stratified by AFP at baseline and relapse. The present study found that patients with positive AFP at baseline and relapse, as well as those who were negative turned positive, had the shortest RDS and OS.

**Conclusions:**

Not only AFP at baseline but also subsequent AFP at relapse can be used to predict a post-recurrence survival, which can help evaluate mortality risk stratification of patients after relapse.

## Introduction

Hepatocellular carcinoma (HCC) ranks sixth in morbidity and third in mortality with a rapidly rising trend in the world, making it one of the most common malignancies (1). In China, the majority of HCC patients are caused by chronic hepatitis B or C virus infection. Therefore, how to reduce the high recurrence and mortality of virus-associated HCC has always been the top priority of research (2, 3).

Alpha fetoprotein (AFP) as a specific tumor marker not only plays an important role in the diagnosis of HCC, but also be used to assess tumor burden, which is why it is used to predict prognosis in patients with HCC after various treatments (4, 5). Some studies have also demonstrated that AFP included can improve the predictive efficacy of prognostic scoring systems for HCC (6, 7). However, most of these study populations are individuals receiving radical hepatectomy and liver transplantation, and a few focus on patients treated with minimally invasive treatment like transcatheter arterial chemoembolization (TACE) plus ablation (hereinafter, combination therapy).

As a potential curative treatment for HCC, locoregional therapy is increasingly accepted by patients and physician at various centers. We urgently need to understand whether the prognostic value of AFP analyzed in previous studies applies to patients receiving locoregional therapy. Therefore, we conducted a five-year prospective cohort study to confirm the prognostic value of AFP at baseline (b-AFP), subsequent AFP at relapse (r-AFP), and AFP alteration in virus-related HCC patients undergoing combination therapy.

## Materials and Methods

### Patients Enrolled

A total of 583 subjects with newly diagnosis of virus-related HCC who were admitted to Beijing You ‘an Hospital, Capital Medical University from January 1, 2012 to December 31, 2016 were prospectively enrolled. Patients were aged between 18 and 75 years and received combination therapy for curative purposes. The diagnosis of HCC conforms to the suggestions of American Association for the Study of Liver Diseases (AASLD) (8). In order to eliminate the negative survival effects of incomplete ablation, only patients with complete ablation were considered. Individuals could not be enrolled if they were in accordance with the following circumstances: 1) Child-Pugh class C; 2) accompanied by other severe disease (other types of tumors or coagulopathy); 3) secondary liver cancer; 4) advanced liver cancer.

Patient information is confidential and, because it is a minimum-risk study, the requirement for informed consent of patients was waived. The study was approved by the ethics committee of Beijing You ‘an Hospital affiliated to Capital Medical University.

### Clinical Data Collection

We collected the laboratory data of patients within 7 days before ablation and at the time of the first recurrence, including the following aspects: 1) demographic data, such as sex, age, history of antiviral therapy, and history of hypertension and diabetes mellitus; 2) tumor information, including AFP, viral load, tumor size (denoted by the maximum diameter), tumor number; 3) HCC etiologies, such as hepatitis B virus (HBV), hepatitis C virus (HCV), co-infection; 4) liver function markers, like Child-Pugh class, cirrhosis; 5) laboratory data, such as alanine aminotransferase (ALT), aspartate aminotransferase (AST), total bilirubin, serum albumin, γ-glutamyl transpeptidase (γ-GT), international normalized ratio (INR); and 6) therapy-related factors, including the ablation modality and times. It should be noted that AFP was collected not only at baseline but also at the time of recurrence to verify whether the dynamic change of AFP affected the long-term prognosis after relapse.

### Therapeutic Procedure

All patients enrolled received TACE combined with locoregional ablation, and the latter was conducted under computed tomography (CT) or magnetic resonance imaging (MRI) guidance within 2 weeks after TACE. The operational steps of the two interventions have been described in detail in the previous articles of our team, so we won’t go into them here (9).

### Follow-Up

Patients included were followed up every 3 to 6 months, including physical examination, blood test, and abdominal CT/MRI. Individuals who relapse were treated with TACE and/or ablation therapies such as radiofrequency ablation (RFA), microwave ablation (MWA), and argon-helium knife cryoablation (AHC).

Recurrence were confirmed as soon as enhanced signals are shown within, near, or outside the primary tumor. Recurrence-free survival (RFS) is delineated as the time from the diagnosis of HCC to the first relapse or death. The overall survival (OS) was defined as the time from the diagnosis of HCC to HCC-associated mortality or the last follow-up. The recurrence-death survival (RDS) was identified as the time from the first recurrence to HCC-related mortality or the last follow-up. The date of the last follow-up for the present study was July 1, 2020.

### Statistical Analysis

Continuous data were expressed as mean ± standard deviation and compared among groups using the analysis of variance. Categorical data were expressed as the frequency and compared using the Chi-square test. The independent risk factors of relapse and mortality were determined by Cox proportional hazard model. The cumulative 1-, 3-, 5-year RFS, OS and RDS rates were computed by Kaplan-Meier method and were compared between groups using the log-rank test. P value <0.05 was considered to be statistically significant. IBM SPSS version 21.0 (SPSS Inc., Chicago, IL, USA) and R Foundation Statistical software (version 3.6.3) were used to process the data involved.

## Results

### Patients Characteristics

A total of 583 patients were enrolled in the study, including 469 (80.4%) men and 114 (19.6%) women. 135 (23.2%) had high blood pressure and 99 (17.0%) had diabetes mellitus. 301 (51.6%) people had taken antiviral drugs. There were 431 (73.9%) patients with Child-Pugh class A and the rest with Child-Pugh class B. Of these patients, 500 (85.5%), 58 (9.9%), and 25 (4.3%) of HCC were associated with HBV, HCV, and co-infection, respectively. There were 304 (52.1%), 150 (25.7%), and 129 (22.1%) individuals who were treated with RFA, MWA, and AHC. And among those, 80 (13.7%) received multiple ablations according to location, extension and size of the tumors. 91% (531/583) of patients who had achieved complete remission after local treatment in this cohort were BCLC stage 0 and A, so there was a significant difference in the number of cases between groups according to BCLC stage, which may lead to biased results. And for this reason, the variable of tumor stage was not analysed.

### Prognostic Outcomes

By the end of follow-up, 58 cases were lost to follow-up. the median OS was not achieved, while the median RFS was 24.5 months. The median follow-up after treatment was 60.2 months (25~75th percentiles, 44.8~82.6 months). A total of 431 (73.9%) patients relapsed and 200 (34.3%) died. The cumulative OS rates of 1, 3, and 5 years were 99.0% (577/583), 86.1% (502/583), and 74.3% (433/583). The corresponding cumulative RFS rates were 72.0% (420/583), 40.1% (234/583), and 30.4% (177/583).

### AFP at Baseline as a Prognostic Factor Associated With RFS, OS

A total of 583 cases were enrolled, of which 3 had missing AFP values. There were 240 subjects with negative AFP and 340 with positive AFP at baseline. The relationship between AFP at baseline and clinicopathological factors was analysed, and we found that male, multiple tumors, low albumin, high AST, high γ-GT, and high viral load were associated with positive AFP with *P* values less than 0.05 (**Table 1**). We analyzed whether b-AFP was an independent risk factor for recurrence and long-term survival using Cox proportion hazard model (**Tables 2**, **3**). The results showed that age, sex, tumor number, tumor size, and AFP were independent risk factors for recurrence, while age, etiology, Child-Pugh class, tumor number, and γ-GT were independently associated with long-term survival. Patients with positive b-AFP had a 24% increased risk of recurrence compared with those who were negative, but there was no effect on overall survival. Patients with negative and positive b-AFP had a median RFS of 32.4 and 21.2 months, with a median OS not reached for the former and 96.5 months for the latter. The cumulative 1-, 3-, 5- year RFS rates of patients who were negative were 80.0% (192/240), 45.8% (110/240), and 33.8% (81/240), and the corresponding rates were 66.5% (226/340), 35.6% (121/340), and 27.6% (94/340) for patients who were positive (P=0.011).

**Table 1:** The association between APF at baseline and clinicopathological indicators.

Variables	Total	Negative	Positive	*P value*
		n = 240	n = 340	
Age (years old)	56.33 ± 8.80	57.21 ± 8.65	55.78 ± 8.82	0.053
Gender (Male/Female)	467/113	205/35	262/78	**0.012**
Hypertension (yes/no)	134/446	51/189	83/257	0.374
Diabetes mellitus (yes/no)	98/482	42/198	56/284	0.745
Antiviral history (yes/no)	300/278	135/104	165/174	0.064
Etiology (HBV/HCV/co-infection)	497/58/25	210/21/9	287/37/16	0.579
Cirrhosis (yes/no)	485/95	194/46	291/49	0.128
Child-Pugh class (A/B)	428/152	186/54	242/98	0.088
Fractional ablation (yes/no)	80/500	27/213	53/287	0.136
Ablative.modality (RFA/MWA/AHC)	302/150/128	127/62/51	175/88/77	0.913
Tumor number (single/multiple)	392/188	183/57	209/131	**<0.001**
Tumor size (≤30mm/>30mm)	403/177	167/73	236/104	0.965
Alanine aminotransferase (U/L)	40.11 ± 27.20	37.48 ± 28.28	41.97 ± 26.29	0.053
Aspartate aminotransferase (U/L)	36.77 ± 19.80	32.88 ± 17.55	39.51 ± 20.83	**<0.001**
Total bilirubin (μmol/L)	19.18 ± 10.06	18.53 ± 9.59	19.64 ± 10.37	0.192
Albumin (g/l)	37.02 ± 4.45	37.71 ± 4.42	36.53 ± 4.41	**0.002**
γ-GT (IU/L)	73.21 ± 58.67	63.67 ± 50.31	79.94 ± 63.12	**0.001**
INR	1.08 ± 0.12	1.07 ± 0.12	1.09 ± 0.12	0.077
Viral load (<1000/1000-20000/>20000IU/mL)	302/118/137	161/35/36	141/83/101	**<0.001**

AFP, alpha fetoprotein; RFA, radiofrequency ablation; MWA, microwave ablation; AHC, argon-helium knife cryoablation; INR, international normalized ratio. Bold values indicate that the P value is less than 0.05, there is statistical difference.

**Table 2:** AFP at baseline as a prognostic factor associated with RFS.

Variables	Univariate		Multivariate	
	HR (95%CI)	*P* value	HR (95%CI)	*P* value
Age (years old)	1.02 (1.00-1.03)	**0.005**	1.02 (1.01-1.03)	**0.004**
Gender (male/female)	1.58 (1.22-2.04)	**0.001**	1.68 (1.29-2.20)	**<0.001**
Hypertension (yes/no)	0.99 (0.79-1.24)	0.918		
Diabetes mellitus (yes/no)	1.06 (0.83-1.36)	0.639		
Antiviral history (yes/no)	0.87 (0.72-1.05)	0.136		
Etiology (HBV/HCV/co-infection)	1.00 (0.83-1.21)	0.985		
Cirrhosis (yes/no)	1.39 (1.06-1.81)	**0.017**	1.34 (1.00-1.79)	0.052
Child-Pugh class (A/B)	1.22 (0.99-1.51)	0.069		
Fractional ablation (yes/no)	1.45 (1.12-1.89)	**0.005**	0.92 (0.69-1.25)	0.605
Ablative modality (RFA/MWA/AHC)	0.94 (0.84-1.06)	0.317		
Tumor number (single/multiple)	1.59 (1.31-1.93)	**<0.001**	1.56 (1.26-1.93)	**<0.001**
Tumor size (≤30mm/>30mm)	1.69 (1.38-2.06)	**<0.001**	1.67 (1.35-2.08)	**<0.001**
Alanine aminotransferase (U/L)	1.00 (1.00-1.01)	0.301		
Aspartate aminotransferase (U/L)	1.01 (1.00-1.01)	**0.001**	1.01 (1.00-1.01)	0.077
Total bilirubin (μmol/L)	1.01 (1.00-1.02)	**0.046**	1.00 (0.99-1.01)	0.939
Albumin (g/L)	0.96 (0.94-0.98)	**<0.001**	0.99 (0.96-1.02)	0.471
γ-GT (IU/L)	1.00 (1.00-1.01)	**<0.001**	1.00 (1.00-1.53)	0.155
INR	2.51 (1.19-5.31)	**0.016**	1.18 (0.41-3.35)	0.761
Alpha fetoprotein (negative/positive)	1.28 (1.06-1.56	**0.012**	1.24 (1.00-1.53)	**0.049**
Viral load (<1000/1000-20000/>20000IU/mL)	1.14 (1.02-1.27)	**0.024**	1.10 (0.92-1.33)	0.295

AFP, alpha fetoprotein; RFS, recurrence-free survival; HR, hazard ratio; RFA, radiofrequency ablation, MWA, microwave ablation; AHC, argon-helium knife cryoablation; INR, international normalized ratio. Bold values indicate that the P value is less than 0.05, there is statistical difference.

**Table 3:** AFP at baseline as a prognostic factor associated with OS.

Variables	Univariate		Multivariate	
	HR (95%CI)	*P* value	HR (95%CI)	*P* value
Age (years old)	1.02 (1.00-1.04)	**0.012**	1.02 (1.01-1.04)	**0.011**
Gender (male/female)	1.48 (1.01-2.19)	**0.046**	1.46 (0.98-2.19)	0.066
Hypertension (yes/no)	0.81 (0.57-1.14)	0.224		
Diabetes mellitus (yes/no)	1.10 (0.76-1.58)	0.610		
Antiviral history (yes/no)	0.76 (0.58-1.01)	0.057		
Etiology (HBV/HCV/Co-infection)	1.37 (1.08-1.73)	**0.010**	1.30 (1.00-1.69)	**0.049**
Cirrhosis (yes/no)	1.32 (0.86-2.00)	0.201		
Child-Pugh class (A/B)	1.82 (1.35-2.44)	**<0.001**	1.47 (1.03-2.11)	**0.034**
Fractional ablation (yes/no)	1.54 (1.07-2.21)	**0.019**	1.06 (0.71-1.58)	0.788
Ablative modality (RFA/MWA/AHC)	1.07 (0.90-1.26)	0.460		
Tumor number (single/multiple)	1.56 (1.18-2.07)	**0.002**	1.47 (1.08-1.99)	**0.013**
Tumor size (≤30mm/>30mm)	1.39 (1.04-1.86)	**0.028**	1.25( 0.90-1.73)	0.182
Alanine aminotransferase (U/L)	1.00 (0.99-1.01)	0.202		
Aspartate aminotransferase (U/L)	1.01 (1.00-1.01)	**0.009**	1.00 (0.99-1.01)	0.710
Total bilirubin (μmol/L)	1.02 (1.00-1.03)	**0.017**	1.00 (0.98-1.01)	0.812
Albumin (g/L)	0.93 (0.90-0.96)	**<0.001**	0.98 (0.94-1.02)	0.394
γ-GT (IU/L)	1.00 (1.00-1.01)	**<0.001**	1.00 (1.00-1.01)	**0.029**
INR	6.31 (2.27-17.52)	**<0.001**	2.87 (0.69-11.98)	0.149
Alpha fetoprotein (negative/positive)	1.15 (0.86-1.53)	0.347		
Viral load (<1000/1000-20000/>20000IU/mL)	1.24 (1.05-1.45)	**0.010**	1.15 (0.97-1.36)	0.115

AFP, alpha fetoprotein; RFS, recurrence-free survival; HR, hazard ratio; RFA, radiofrequency ablation, MWA, microwave ablation; AHC, argon-helium knife cryoablation; INR, international normalized ratio. Bold values indicate that the P value is less than 0.05, there is statistical difference.

### AFP at Relapse as a Prognostic Factor Related to RDS

**Figure 1:**
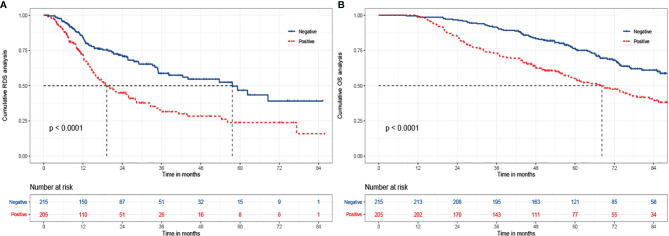
The Kaplan-Meier analysis of RDS and OS for patients based on AFP at relapse. **(A)** is the Kaplan-Meier analysis of RDS; **(B)** is the Kaplan-Meier analysis of OS. RDS, recurrence-death survival; OS, overall survival.

Of the 580 cases, 431 recurred and AFP values could not be obtained in 11 cases. So, there were 215 subjects with negative AFP and 205 with positive AFP at the time of recurrence. We continued to explore the relationship between r-AFP and long-term survival after recurrence using Cox proportion hazard model (**Table 4**). The findings showed that antiviral history, Child-Pugh class, tumor number, tumor size, γ-GT, and AFP were adverse parameters for post-recurrence survival. Individuals with positive r-AFP had a 68% increased risk of death compared with those with negative r-AFP (P=0.002). Only 72 (33.5%) of the 215 patients with negative r-AFP died, while 106 (51.7%) of the 205 with positive r-AFP died (<0.001). The median RDS of two groups were 57.6 and 19.3 months, respectively. The cumulative RDS rates for 1, 3, 5 years in patients who were negative were 85.6% (184/215), 70.2% (151/215), and 67.4% (145/215), while the corresponding rates were 75.1% (154/205), 51.2% (105/205), and 48.8% (100/205) in those who were positive (P<0.001). The cumulative 1-, 3-, 5-year OS rates for the former were 99.1% (213/215), 91.6% (197/215), and 78.1% (168/215), while for the latter were 98.5% (202/205), 73.7% (151/205), and 58.5% (120/205) (P<0.001) (**Figure 1**).

**Table 4:** AFP at relapse as a prognostic factor associated with RDS.

Variables	Univariate		Multivariate	
	HR (95%CI)	*P* value	HR (95%CI)	*P* value
Age (years old)	1.01 (0.99-1.03)	0.414		
Gender (male/female)	0.68 (0.44-1.05)	0.082		
Hypertension (yes/no)	0.78 (0.54-1.12)	0.180		
Diabetes mellitus (yes/no)	1.05 (0.71-1.55)	0.790		
Antiviral history (yes/no)	0.72 (0.54-0.96)	**0.025**	0.72 (0.54-0.98)	**0.035**
Etiology (HBV/HCV/Co-infection)	1.04 (0.80-1.36)	0.750		
Cirrhosis (yes/no)	1.31 (0.85-2.04)	0.222		
Child-Pugh class (A/B)	1.51 (1.11-2.06)	**0.009**	1.45 (1.05-2.00)	**0.024**
Fractional ablation (yes/no)	1.61 (1.12-2.33)	**0.011**	1.06 (0.70-1.61)	0.788
Ablative modality (RFA/MWA/AHC)	1.03 (0.87-1.23)	0.706		
Tumor number (single/multiple)	1.63 (1.21-2.18)	**0.001**	1.45 (1.05-2.00)	**0.024**
Tumor size (≤30mm/>30mm)	1.53 (1.13-2.07)	**0.006**	1.39 (1.00-1.94)	**0.050**
Alanine aminotransferase (U/L)	1.01 (1.00-1.01)	**0.010**	1.00 (1.00-1.01)	0.517
Aspartate aminotransferase (U/L)	1.01 (1.00-1.01)	**0.001**	1.00 (1.00-1.01)	0.522
Total bilirubin (μmol/L)	1.00 (1.00-1.01)	0.850		
Albumin (g/L)	0.98 (0.96-1.00)	0.093		
γ-GT (IU/L)	1.00 (1.00-1.01)	**<0.001**	1.00 (1.00-1.01)	**0.007**
Alpha fetoprotein (negative/positive)	2.12 (1.57-2.86)	**<0.001**	1.68 (1.22-2.32)	**0.002**

AFP, alpha fetoprotein; RFS, recurrence-free survival; HR, hazard ratio; RFA, radiofrequency ablation, MWA, microwave ablation; AHC, argon-helium knife cryoablation. Bold values indicate that the P value is less than 0.05, there is statistical difference.

### The Association of Prognosis With b-AFP and Subsequent AFP

**Figure 2:**
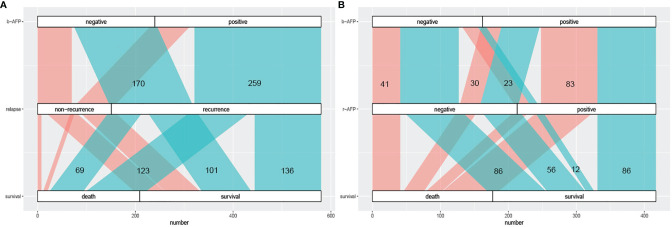
The Sankey diagram of correlation between AFP prognosis. **(A)** the correlation between AFP at baseline and prognosis including recurrence and long-term survival; cyan part represents the recurrence group and coral part represents the non-recurrence group. **(B)** the correlation between AFP alteration (at baseline and relapse) and long-term survival; cyan part represents the survival group and coral part represents the death group. AFP, alpha fetoprotein; b-AFP, AFP at baseline; r-AFP, AFP at relapse.

We plotted the Sankey diagram to visually represent the relationship between AFP and outcomes of HCC patients who received combination therapy (**Figure 2**). As can be seen in **Figure 2A**, 259 (76.2%) of the 340 patients with positive b-AFP relapsed, and of those who relapse, 123 (47.5%) died. Similarly, of 240 subjects who were negative, 170 (70.8%) relapsed, and of those who relapse, 69 (40.6%) died. As shown in **Figure 2B**, 35 (21.6%) of the 162 patients with negative b-AFP turned positive at the time of recurrence, and of this subset, only 12 (34.3%) survived. Of the 255 patients with positive b-AFP, 86 (33.7%) turned negative at the time of relapse, and of this subset, only 30 (34.9%) died.

### The Analysis of Prognostic Data Based on AFP Alteration

We divided patients into four groups based on AFP alteration at baseline and relapse, including N-N (negative at baseline and negative at relapse), N-P (negative at baseline and positive at relapse), P-N (positive at baseline and negative at relapse), and P-P (positive at baseline and positive at relapse), and prognostic data were compared pairwise (**Table 5**). Among patients with negative b-AFP, those who turned positive at recurrence had a 130% increased risk of death compared with those who remained negative AFP at relapse (P=0.001). Subjects with positive AFP both at baseline and recurrence had a 125% increased risk of death compared with those who were negative AFP both at baseline and relapse (P<0.001). The post-recurrence survival of patients who turned from positive to negative was significantly better than that of patients who turned from negative to positive (P=0.01).

**Table 5:** The association of prognostic data with AFP alteration.

Groups	RDS			
**OS**	**N-N**	0.001	0.603	<0.001
		2.30 (1.38-3.83)	1.14 (0.71-1.82)	2.25 (1.54-3.27)
	<0.001	**N-P**	0.01	0.875
	3.02 (1.81-5.03)			
	0.958	<0.001	**P-N**	0.002
	1.02 (0.64-1.64)			
	0.001	0.068	0.004	**P-P**
	1.91 (1.32-2.78)			

AFP, alpha fetoprotein; N-N, negative AFP at baseline and negative AFP at relapse; N-P, negative AFP at baseline and positive AFP at relapse; P-N, positive AFP at baseline and negative AFP at relapse; P-P, positive AFP at baseline and positive AFP at relapse.

**Figure 3:**
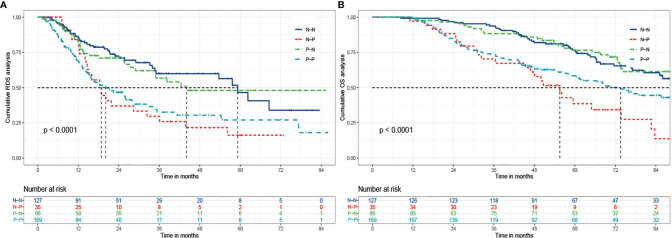
The Kaplan-Meier analysis of RDS and OS for patients based on AFP alteration. **(A)** is the Kaplan-Meier analysis of RDS; **(B)** is the Kaplan-Meier analysis of OS. RDS, recurrence-death survival; OS, overall survival; N-N, negative AFP at baseline and negative AFP at relapse; N-P, negative AFP at baseline and positive AFP at relapse; P-N, positive AFP at baseline and negative AFP at relapse; P-P, positive AFP at baseline and positive AFP at relapse.

Survival curves were plotted for four groups including N-N, N-P, P-N, and P-P, in which 32.3% (41/127), 65.7% (23/35), 34.9% (30/86), and 49.1% (83/169) died, respectively (P<0.001) (**Figure 3**). The cumulative 1-, 3-, and 5- year RDS rates were 86.6% (110/127), 71.7% (91/127), and 69.3% (88/127) in N-N group; 85.7% (30/35), 42.9% (15/35), and 34.3% (12/35) in N-P group; 84.9% (73/86), 68.6% (59/86), and 65.1% (56/86) in P-N group; and 72.8% (123/169), 52.7% (89/169), and 51.5% (87/169) in P-P group (P<0.001). There were also significant differences of OS among the four groups (99.2%, 93.7%, 78.7% vs 97.1%, 71.4%, 45.7% vs 98.8%, 88.4%, 77.9% vs 98.8%, 74.0%, 60.9%, P<0.001)

### The Analysis of Clinicopathological Characteristics Based on AFP Alteration

**Figure 4:**
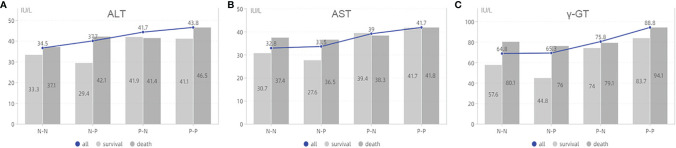
The analysis of laboratory data based on AFP alteration. **(A)** is the association between ALT and survival status based on groups; **(B)** is the association between AST and survival status based on groups; **(C)** is the association between γ-GT and survival status based on groups. ALT, alanine aminotransferase; AST, aspartate aminotransferase;γ-GT, γ-glutamyl transpeptidase; N-N, negative AFP at baseline and negative AFP at relapse; N-P, negative AFP at baseline and positive AFP at relapse; P-N, positive AFP at baseline and negative AFP at relapse; P-P, positive AFP at baseline and positive AFP at relapse.

We further analyzed the relationship between the four groups and clinicopathological characteristics (**Table 6**). The results showed that there were the highest death rates in N-P and P-P groups. There patients generally had multiple tumors with large diameter, which is also why those tended to receive multiple ablations for the purpose of complete response (P<0.05). For laboratory data, patients in the P-P group had higher AST, ALT, gamma-GT, and viral load (P<0.05, **Figure 4**).

**Table 6:** The association of AFP alteration with demographic data.

Variables	Total	N-N	N-P	P-N	P-P	*P value*
		n=127	n=35	n=86	n=169	
Death number	177	41 (32.3%)	23 (65.7%)	30 (34.9%)	83 (49.1%)	**<0.001**
Age (years old)	56.93 ± 8.19	58.07 ± 7.36	56.14 ± 8.22	57.35 ± 8.45	56.01 ± 8.58	0.162
Gender (male/female)	350/67	110/17	31/4	68/18	141/28	0.428
Hypertension (n/%)	98 (23.5%)	32 (25.2%)	6 (17.1%)	21 (24.4%)	39 (23.1%)	0.790
Diabetes mellitus (n/%)	72 (17.3%)	22 (17.3%)	6 (17.1%)	17 (19.8%)	27 (16.0%)	0.902
Antiviral history (n/%)	210 (50.6%)	70 (55.6%)	20 (57.1%)	45 (52.9%)	75 (44.4%)	0.200
Etiology (HBV/HCV/Co-infection)	358/42/17	116/9/2	2028/4/3	1973/8/5	141/21/7	0.303
Cirrhosis (n/%)	361 (86.6%)	111 (87.4%)	27 (77.1%)	72 (83.7%)	151 (89.3%)	0.215
Child-Pugh class (A/B)	302/115	94/33	28/7	61/25	119/50	0.658
Fractional ablation (n/%)	361 (86.6%)	114/13	30/5	77/9	131/38	**0.014**
Ablative modality (RFA/MWA/AHC)	221/107/89	71/26/30	16/13/6	43/24/19	91/44/34	0.574
Tumor number (single/multiple)	261/156	93/34	23/12	56/30	89/80	**0.003**
Tumor size (≤30mm/>30mm)	274/143	89/38	17/18	62/24	106/63	**0.049**
Alanine aminotransferase (U/L)	40.03 ± 25.84	34.53 ± 23.23	37.73 ± 28.75	41.73 ± 23.99	43.78 ± 27.40	**0.018**
Aspartate aminotransferase (U/L)	37.77 ± 20.76	32.83 ± 17.81	33.46 ± 18.32	39.01 ± 20.18	41.74 ± 22.73	**0.002**
Total bilirubin (μmol/L)	19.58 ± 10.33	19.96 ± 11.17	16.83 ± 7.04	19.59 ± 10.08	19.85 ± 10.38	0.432
Albumin (g/L)	36.69 ± 4.39	37.44 ± 4.29	37.09 ± 4.43	36.54 ± 4.59	36.12 ± 4.30	0.072
γ-GT (IU/L)	76.85 ± 61.91	64.84 ± 50.96	65.29 ± 51.93	75.77 ± 63.88	88.81 ± 68.20	**0.006**
INR	1.08 ± 0.12	1.08 ± 0.13	1.06 ± 0.08	1.09 ± 0.13	1.09 ± 0.12	0.538
Viral load (<1000/1000-20000/>20000IU/mL)	210/93/102	88/20/16	20/8/7	34/26/23	68/39/56	**<0.001**

AFP, alpha fetoprotein; N-N, negative AFP at baseline and negative AFP at relapse; N-P, negative AFP at baseline and positive AFP at relapse; P-N, positive AFP at baseline and negative AFP at relapse; P-P, positive AFP at baseline and positive AFP at relapse; RFA, radiofrequency ablation, MWA, microwave ablation; AHC, argon-helium knife cryoablation; INR, international normalized ratio. Bold values indicate that the P value is less than 0.05, there is statistical difference.

## Discussion

Therapeutic treatments for HCC mainly include surgical resection, liver transplantation, locoregional ablation, systemic therapies, etc (10). For patients with early-stage HCC, that is, with a single tumor less than 3cm or with a number of tumors less than 3, RFA can produce comparable 5-year overall survival compared with surgical resection (11). TACE combined with ablation has been shown to be more effective than either treatment alone (12, 13). In the study population receiving combination therapy, only 200 of 583 subjects died during the 8-year follow-up period, with a 5-year survival rate of 74.3%. Ablation therapy has the advantages of repeatable, minimally invasive and rapid recovery. Therefore, patients with large or multiple tumors can be treated with multiple ablations according to the tumor conditions to achieve complete response. Our study has consistently concluded that multiple ablations don’t increase the risk of recurrence or mortality compared with ablation in one session. In conclusion, the efficacy of TACE combined with ablation can be further validated in multiple centers, so as to improve the quality of life and prolong the overall survival of more HCC patients.

With a 5-year recurrence rate of up to 60%, management and treatment of HCC after relapse is challenging (14). So far, only a few studies have explored the management and monitoring of HCC after recurrence (15, 16). No one study has systematically elucidated the relationship between b-AFP, subsequent r-AFP, and AFP alteration and overall survival in patients with HCC receiving locoregional therapy aimed for complete response. In addition, the majority of HCC patients are caused by virus infection, so for the first time, our team examined the prognostic value of AFP among them with virus-related HCC.

We found that male, multiple tumors, low albumin, high AST, high γ-GT, and high viral load were associated with positive AFP, suggesting that patients with positive AFP may tend to have poorer liver function and higher tumor aggressiveness. Our study showed that patients with positive b-AFP had an increased risk of recurrence compared with those with negative AFP, which is consistent with Yang’s findings (17, 18). We further explored the association of r-AFP with post-recurrence survival. As expected, patients with positive r-AFP had an increased risk of death after relapse compared with those who were negative. It can be seen that r-AFP has a better prognostic value than b-AFP, which is in agreement with Tabrizian’s study with a population of HCC patients receiving resection (19).

Interestingly, the study found that patients with positive AFP at baseline and relapse, as well as those who were negative turned positive, had the shortest RDS and OS. These patients were characterized by multiple tumors, large tumors, and elevated transaminases that represent liver damage, suggesting high tumor invasiveness and poor liver function, resulting in a poor prognosis. These findings were consistent with the Tabrizian’s data (15). Therefore, in clinical practice, we should pay close attention not only to patients with positive b-AFP and r-AFP, but also to patients who are negative at baseline but turned positive at the time of relapse, adjusting their follow-up strategies or even taking therapeutic measures, such as TACE, to improve their long-term outcomes after recurrence.

Many studies have reported that patients with negative b-AFP have a better RFS or OS than those with positive b-AFP. Among patients with negative b-AFP, however, our results proved that those who turned positive at the time of recurrence had a worse outcome than those who remained negative at the time of recurrence (20, 21). At present, no relevant studies have reported the mechanism of the change of AFP from negative to positive, but it fully reflects the large heterogeneity of HCC. In clinical practice, we need to develop personalized strategies of the management and treatment for patients with HCC, such as additional diagnostic CT/MRI or follow-up at shorter intervals for patients at higher risk of recurrence and death.

There are several limitations to our study. First of all, this is a single-center study with a large sample size, diversity in treatment experience and preference of physicians in different centers can contribute to different long-term outcomes of HCC patients. Secondly, we did not collect AFP values of patients without recurrence. However, after analysis, the risk of death in patients with recurrence is 328% higher than that in patients without recurrence. Therefore, we should pay more attention to patients with recurrence and focus on their survival after recurrence.

In general, some scholars have only studied the relationship between AFP at baseline and prognosis (20, 21), some have focused on AFP of pre- and post-treatment, that is, AFP response (22–24), and some have only explored the prognostic value of AFP at the time of recurrence (16, 25). Our team is the first to conduct a large prospective cohort study with follow-up of more than 5 years to systematically examine the relationship between AFP from baseline to relapse and survival after recurrence, which can better guide clinical practice. Not all patients with positive b-AFP have a poor prognosis, and not all patients with negative b-AFP have a good prognosis. Thus, HCC patients are managed and followed up based on b-AFP and subsequent AFP at relapse. For patients with negative b-AFP, regular monitoring is still required, and for patients with positive r-AFP, more complete treatment must be taken regardless of AFP at baseline. For example, the post-treatment follow-up interval should be shortened from every six months to every three months in N-P and P-P groups, and adjuvant therapy should be performed when necessary to prolong the prognosis of patients. In summary, we are able to predict recurrence after treatment based on b-AFP and to predict long-term survival after recurrence based on r-AFP and AFP alteration.

## Conclusions

Approximately 60% of HCC patients relapse after radical therapy. AFP at baseline and relapse can be used to predict a long-term survival after recurrence, which can help evaluate mortality risk stratification of patients after recurrence.

## Data Availability Statement

The original contributions presented in the study are included in the article/**Supplementary Material**. Further inquiries can be directed to the corresponding authors.

## Ethics Statement

The studies involving human participants were reviewed and approved by the Ethics Committee of Capital Medical University affiliated Beijing Youan Hospital (No.LL-2019-004-K). Written informed consent for participation was not required for this study in accordance with the national legislation and the institutional requirements.

## Author Contributions

Conceived and designed the protocol: YZ and JZ. Collected data: BL and WQ. Wrote the manuscript: QW. Analyzed data: CZ and JLo. Critically revised and approved the final version of manuscript: CH, JLi, and CY. All authors contributed to the article and approved the submitted version.

## Funding

This study was funded by a grant Beijing Municipal Science & Technology Commission (Z171100001017078), Beijing Key Laboratory (BZ0373), Beijing Municipal Administration of Hospitals’ Ascent Plan (DFL20181701), Beijing Municipal Natural Science Foundation (7191004 and 7202069), Key medical professional development plan of Beijing municipal administration of hospitals (ZYLX201711), and Capital health development project (2020-1-2182 and 2020-2-1153).

## Conflict of Interest

The authors declare that the research was conducted in the absence of any commercial or financial relationships that could be construed as a potential conflict of interest.

## Publisher’s Note

All claims expressed in this article are solely those of the authors and do not necessarily represent those of their affiliated organizations, or those of the publisher, the editors and the reviewers. Any product that may be evaluated in this article, or claim that may be made by its manufacturer, is not guaranteed or endorsed by the publisher.
